# Supplementary material for: Guideline adherence in speech and language therapy in stroke aftercare. A health insurance claims data analysis
Source: PLoS One. 2022 Feb 3;17(2):e0263397. doi: 10.1371/journal.pone.0263397 (PMC8812973; doi:10.1371/journal.pone.0263397)
Supplement: S1 Table — (DOCX) [file pone.0263397.s001.docx]

Supplementary Table 1: Overview of guidelines included in the parameter construction

| **Editor** | **Name of guideline** | **Weblink** | **Date of publication** | **Disease** |
| --- | --- | --- | --- | --- |
| Australian Stroke Foundation (ASF) | Australian Clincial Guidelines for Stroke Management 2017 | https://www.clinicalguidelines.gov.au/portal/2585/clinical-guidelines-stroke-management-2017 | 09/2017, valid until 11/2024 | Stroke |
| Royal College of Physicians (RCP) | Royal College of Physicians Intercollegiate Stroke Working Party | https://www.rcplondon.ac.uk/guidelines-policy/stroke-guidelines | 10/2016, valid: currently | Stroke |
| Scottish Intercollegiate Guideline Network (SIGN) | SIGN: Management of patients with stroke, 118 | https://www.sign.ac.uk/media/1056/sign118.pdf | 06/2010, valid: currently | Stroke |
| German Society of Neurology (DGN-A) | Rehabilitation of aphasic disorders after stroke | https://dgn.org/leitlinien/ll-92-2012-rehabilitation-aphasischer-stoerungen-nach-schlaganfall/ | 30.09.2012, valid until 29.09.2017 (currently under revision) | Aphasia |
| German Society of Neurology (DGN-D) | Neurogenic speech disorders (dysarthria) | https://www.awmf.org/leitlinien/detail/ll/030-103.html | 06/2018, valid until 31.12.2020 | Dysarthria |
| Aphasie Suisse (AP) | Recommendations for the treatment of aphasia | http://www.aphasie.org/de/3-fachpersonen/3.4-guidelines/empfehlungen-zur-behandlung-von-aphasien-d.pdf | 2006 | Aphasia |
| Society for Aphasia Research and Treatment (GAB) and German Society for Neurotraumatology and Clinical Neurorehabilitation (DGNKN) | Quality criteria and standards for the treatment of patients with acquired neurogenic disorders of language (aphasia) and speech (dysarthria) | https://www.aphasiegesellschaft.de/wp-content/uploads/2019/02/LL_2000_GAB_DGNKN.pdf | 2002 | Aphasia, Dysarthria |
| German Society for General and Family Medicine (DEGAM) | Stroke | https://www.awmf.org/leitlinien/detail/ll/053-011.html | 02/2020, valid until 02/2025 | Aphasia, Dysarthria |
| German Society for Phoniatrics and Pedaudiology (DGPP) | Functional diagnostics and therapy of communication disorders in adult neurogenic speech and voice disorders | https://dgpp.de/cms/media/download_gallery/LL_Neurogene_Sprech-_und_Stimmstoerungen.pdf | 2012 | Dysarthria |
