# Supplementary material for: Guideline adherence in speech and language therapy in stroke aftercare. A health insurance claims data analysis
Source: PLoS One. 2022 Feb 3;17(2):e0263397. doi: 10.1371/journal.pone.0263397 (PMC8812973; doi:10.1371/journal.pone.0263397)
Supplement: Attachment — Submitted filename: Rebuttal letter_R2.docx [file pone.0263397.s003.docx]

PONE-D-21-17814R1

Guideline adherence in speech and language therapy in stroke aftercare. A health insurance claims data analysis.

PLOS ONE

Dear Professor Jetté,

Thank you very much for reviewing our manuscript again and giving us the opportunity to revise some minor issues with the help of reviewer #1. We discussed the comments in the authors' group and incorporated them point by point into the manuscript. Our answers and new text parts are in blue font.

Kind regards

Dr. Daniel Schindel

*********************

Dear Dr. Schindel,

Thank you for submitting your manuscript to PLOS ONE. After careful consideration, we feel that it has merit but does not fully meet PLOS ONE’s publication criteria as it currently stands. Therefore, we invite you to submit a revised version of the manuscript that addresses the points raised during the review process.

Specifically, please respond to Reviewer 1's revision requests.

Please submit your revised manuscript by Jan 28 2022 11:59PM. If you will need more time than this to complete your revisions, please reply to this message or contact the journal office at [plosone@plos.org](mailto:plosone@plos.org). When you're ready to submit your revision, log on to <https://www.editorialmanager.com/pone/> and select the 'Submissions Needing Revision' folder to locate your manuscript file.

Please include the following items when submitting your revised manuscript:

- A rebuttal letter that responds to each point raised by the academic editor and reviewer(s). You should upload this letter as a separate file labeled 'Response to Reviewers'.
- A marked-up copy of your manuscript that highlights changes made to the original version. You should upload this as a separate file labeled 'Revised Manuscript with Track Changes'.
- An unmarked version of your revised paper without tracked changes. You should upload this as a separate file labeled 'Manuscript'.

If you would like to make changes to your financial disclosure, please include your updated statement in your cover letter. Guidelines for resubmitting your figure files are available below the reviewer comments at the end of this letter.

If applicable, we recommend that you deposit your laboratory protocols in protocols.io to enhance the reproducibility of your results. Protocols.io assigns your protocol its own identifier (DOI) so that it can be cited independently in the future. For instructions see: <https://journals.plos.org/plosone/s/submission-guidelines#loc-laboratory-protocols>. Additionally, PLOS ONE offers an option for publishing peer-reviewed Lab Protocol articles, which describe protocols hosted on protocols.io. Read more information on sharing protocols at <https://plos.org/protocols?utm_medium=editorial-email&utm_source=authorletters&utm_campaign=protocols>.

We look forward to receiving your revised manuscript.

Kind regards,

Marie Jetté
Academic Editor
PLOS ONE

[Note: HTML markup is below. Please do not edit.]

Reviewers' comments:

Reviewer's Responses to Questions

**Comments to the Author**

1. If the authors have adequately addressed your comments raised in a previous round of review and you feel that this manuscript is now acceptable for publication, you may indicate that here to bypass the “Comments to the Author” section, enter your conflict of interest statement in the “Confidential to Editor” section, and submit your "Accept" recommendation.

Reviewer #1: All comments have been addressed

Reviewer #2: All comments have been addressed

2. Is the manuscript technically sound, and do the data support the conclusions?

The manuscript must describe a technically sound piece of scientific research with data that supports the conclusions. Experiments must have been conducted rigorously, with appropriate controls, replication, and sample sizes. The conclusions must be drawn appropriately based on the data presented.

Reviewer #1: Yes

Reviewer #2: (No Response)

3. Has the statistical analysis been performed appropriately and rigorously?

Reviewer #1: Yes

Reviewer #2: (No Response)

4. Have the authors made all data underlying the findings in their manuscript fully available?

The [PLOS Data policy](http://www.plosone.org/static/policies.action#sharing) requires authors to make all data underlying the findings described in their manuscript fully available without restriction, with rare exception (please refer to the Data Availability Statement in the manuscript PDF file). The data should be provided as part of the manuscript or its supporting information, or deposited to a public repository. For example, in addition to summary statistics, the data points behind means, medians and variance measures should be available. If there are restrictions on publicly sharing data—e.g. participant privacy or use of data from a third party—those must be specified.

Reviewer #1: Yes

Reviewer #2: (No Response)

5. Is the manuscript presented in an intelligible fashion and written in standard English?

PLOS ONE does not copyedit accepted manuscripts, so the language in submitted articles must be clear, correct, and unambiguous. Any typographical or grammatical errors should be corrected at revision, so please note any specific errors here.

Reviewer #1: No

Reviewer #2: (No Response)

6. Review Comments to the Author

Please use the space provided to explain your answers to the questions above. You may also include additional comments for the author, including concerns about dual publication, research ethics, or publication ethics. (Please upload your review as an attachment if it exceeds 20,000 characters)

Reviewer #1: Manuscript #: PONE-D-21-17814R1

Title: Guideline adherence in speech and language therapy in stroke aftercare. A health insurances claims data analysis

Article type: Research Article

Summary
The authors have carefully responded to the reviewer comments and revised their manuscript accordingly. There are some additional minor concerns that have been identified (below) that could be addressed. Additionally, there are several remaining grammatical issues. It would likely improve the readability of the manuscript if it was proofread/revised in its entirety for English grammar again. If these minor issues are addressed satisfactorily, it is likely that this manuscript could be accepted.

Dear Reviewer,

Thank you for rereading and commenting on our manuscript. Your detailed hints were again very helpful and useful to revise the paper. The authors discussed the points you mentioned and implemented them as shown below. We have also had the manuscript proofread again by a professional proof reading service.

Best wishes
The authors

Abstract
In the sentence that starts “Treatment oriented on medical guidelines…”, the word “medical” could be misleading, as it often refers to medication. Perhaps this could be changed to “evidence-based guidelines” or something similar.

Answer (AW): We have changed the wording as you suggested.

Revised sentence: “Treatment oriented on evidence-based guidelines seems likely to improve outcomes.”

As Reviewer 2 points out, using hospitalization duration as a proxy for stroke severity is not ideal. To make the abstract more clearly represent the data, please revise the sentence that starts “Older age and increasing severity of stroke…” by replacing “severity of stroke” with “hospitalization duration”.

AW: We have changed that.

Revised sentence: “Older age and longer hospitalization duration increased the likelihood of guideline recommendations being implemented and earlier initiation of stroke aftercare measures.”

The abstract seems to be missing some of the most salient findings of the paper. Please consider including in the Results section of the Abstract that of the 90.3% of post-stroke patients with an SLT diagnosis, only 44.1% received outpatient SLP services within the first year post-stroke.

AW: Thank you for pointing on that. We included the findings as follows.

New sentences: “Within the first year after the stroke, 90.3% of patients were diagnosed with speech impairments and swallowing disorders. Overall, 44.1% of patients received outpatient speech and language therapy aftercare.”

Methods

In the Guideline parameters to test for adherence section: From the sentence that starts “Extraction of parameters”, please delete “were involved” from the end of the sentence (Lines 119-121). Also, please add a comma after “medical doctor”.

AW: Thank you. We changed that.

Revised sentence: “Extraction of parameters to test adherence using claims data was conducted by three scientific researchers: an experienced speech therapist, a medical doctor, and a medical sociologist.”

In the Guideline parameters to test for adherence section: Please add a colon after the sentence that starts “In total, five specific testable…” Otherwise, the sentence that follows is hard to interpret. (Lines 125-126).

AW: We added a colon after the sentence to make clear that a kind of enumeration follows.

Revised sentence: “In total, five specific testable parameters for SLT provision were extracted from the guidelines (Table 1): Patients showing speech and language disorders should be treated by professional speech and language therapists (Parameter 1: speech therapists).”

In the Guideline parameters to test for adherence section: It is still difficult to interpret the sentence that includes, “we defined having at least two specific diagnoses of speech disturbance ...” (Lines 127 – 131). Perhaps you could add the specific ICD-10-GM codes for aphasia and dysarthria (R47.01?) and separate these two conditions in the sentence (e.g., “aphasia (ICD-10-GM code R47.X), dysarthria (ICD-10-GM code R47.X”)) so that the sentence is clearer. Presently, it looks like dysarthria and aphasia have the same ICD-10 code.

AW: We revised that part. For the selection of the data we used the more general three-digit diagnostic code "R47". This includes the diagnostic codes R47.0 (aphasia), R47.1 (dysarthria), and R47.8 (other and unspecified speech and language disorders). We hope that this is now clearer.

New sentence: “To account for spontaneous remission and early inpatient therapy of disorders [22-24], we defined having at least two specific diagnoses of speech disturbance (ICD-10-GM code R47, including aphasia (ICD-10-GM code R47.0), dysarthria (ICD-10-GM code R47.1), and “other and unspecified speech and language disorders” (ICD-10-GM code R47.8)) or swallowing difficulties (dysphagia (ICD-10-GM code R13)) during inpatient or outpatient care as the prerequisite for determining a need of aftercare SLT.”

Results

The last sentence of the Patients with SLT subsection needs to be revised (“Patients receiving aftercare SLT were more frequently and for a longer period in rehabilitation measures after the initial stroke”). (Lines 204-205)

AW: We have divided the sentence into two sentences.

Revised sentences: “In addition, patients who were in inpatient rehabilitation after stroke were more likely to get SLT aftercare. With increasing rehabilitation duration, the likelihood of receiving SLT also increased.”

Discussion

In the second sentence of the first paragraph of the Discussion, it would be helpful to clarify that hospitalization duration was used as a proxy for stroke severity by putting this information in parentheses (i.e., “patients with severe stroke (as captured by hospitalization duration)”). (Lines 256 - 258).

AW: We have added the operationalization of severity in parentheses.

Please make the fourth sentence of the first paragraph of the Discussion section clearer (“Younger patients needed more time for the take-up of aftercare speech therapies.”) (Line 259-260)

AW: We divided the sentence into two sentences and revised them.

Revised sentences: “In the case of younger patients, more time elapsed before the take-up of aftercare speech therapies. In addition, patients of older age were less likely to use SLT aftercare than younger patients.”

Please revise the last sentence of the first paragraph of the Discussion section that starts “A final evaluation…” (Lines 261-262). Perhaps you could start the sentence with, “A deeper look into the data shows that …”, or something similar that hints to the reader what will be discussed in the remainder of the Discussion.

AW: Thank you for your suggestion. We divided the sentence into two sentences.

Revised sentences: “A deeper look into the data shows that the implementation of recommendations is not necessarily related to specific patient groups per se. While some recommendations are implemented well, others for the same group might appear to be in great need of improvement.”

In the first sentence of the Speech therapists subsection of the Discussion, do you mean “multiple diagnoses” rather than “repeated diagnosis”? Could you add swallowing impairments to this sentence?

AW: Thank you. We revised that.

Revised sentence: “The majority of patients with multiple diagnosis of specific speech, language, and swallowing impairments receives aftercare through speech and language therapists.”

In the first paragraph of the Speech therapists subsection of the Discussion, please revise “Patients with less severe stroke or first stroke” to “Patients with less severe stroke or following an initial stroke”. (Lines 285-286)

AW: We changed that.

Revised sentence: “Patients with less severe stroke or following an initial stroke less frequently received SLT aftercare, or made use of such a therapy, despite a relevant diagnosis.”

In the first paragraph of the Early Initiation and continuity subsection of the Discussion, please change “therapy resumption” in the first sentence to “therapy initiation.” (Line 324)

AW: Thank you. We changed that.

New sentence: “One aspect of guideline-adherent therapy after stroke is early therapy initiation for stroke patients after hospital discharge.”

Tables

On Table 2, it might be helpful to revise the 7th row description from “Speech, language disorders: R47, R13” to “Dysarthria, aphasia, or dysarthria” (and add the relevant ICD-10 codes). Please include the raw number and percentage of the population for each of the three codes in the table (e.g., How many individuals had dysphagia?).

AW: Thank you for your suggestion. We have adjusted the labeling in the table and added the footnote to row 7 regarding the diagnosis codes. Unfortunately, a breakdown for the individual diagnoses is no longer possible. We really would have liked to have the individual disorder diagnoses reported separately. But, the data were aggregated in the course of data preparation and various diagnoses were combined. The aggregation of the data was a requirement of the data donors (health insurance companies) because this makes it more difficult to re-identify individual patients. We regret not being able to implement this advice.

Reviewer #2: (No Response)

7. PLOS authors have the option to publish the peer review history of their article ([what does this mean?](https://journals.plos.org/plosone/s/editorial-and-peer-review-process#loc-peer-review-history)). If published, this will include your full peer review and any attached files.


If you choose “no”, your identity will remain anonymous but your review may still be made public.


**Do you want your identity to be public for this peer review?** For information about this choice, including consent withdrawal, please see our [Privacy Policy](https://www.plos.org/privacy-policy).

Reviewer #1: No

Reviewer #2: No

[NOTE: If reviewer comments were submitted as an attachment file, they will be attached to this email and accessible via the submission site. Please log into your account, locate the manuscript record, and check for the action link "View Attachments". If this link does not appear, there are no attachment files.]

While revising your submission, please upload your figure files to the Preflight Analysis and Conversion Engine (PACE) digital diagnostic tool, <https://pacev2.apexcovantage.com/>. PACE helps ensure that figures meet PLOS requirements. To use PACE, you must first register as a user. Registration is free. Then, login and navigate to the UPLOAD tab, where you will find detailed instructions on how to use the tool. If you encounter any issues or have any questions when using PACE, please email PLOS at [figures@plos.org](mailto:figures@plos.org). Please note that Supporting Information files do not need this step.

*In compliance with data protection regulations, you may request that we remove your personal registration details at any time. [(Remove my information/details)](https://www.editorialmanager.com/pone/login.asp?a=r). Please contact the publication office if you have any questions.*
